# Whole-genome sequencing and annotation of *Micrococcus luteus* TG3.30 isolated from sugarcane plantation soil in São Paulo, Brazil

**DOI:** 10.1128/mra.01073-25

**Published:** 2026-02-03

**Authors:** Lara Maria Biancheti, Nathália Vilela, Eleni Gomes, Lisbeth Olsson

**Affiliations:** 1 Laboratory of Biochemistry and Applied Microbiology, Department of Biology, São Paulo State University 28108, São José do Rio Preto, São Paulo, Brazil; 2 Institute for Research in Bioenergy (IPBen), São Paulo State University 28108, São José do Rio Preto, São Paulo, Brazil; 3 Department of Life Sciences, Division of Industrial Biotechnology, Chalmers University of Technology 11248, Gothenburg, Sweden; DOE Joint Genome Institute, Berkeley, California, USA

**Keywords:** *Micrococcus luteus*, whole-genome sequence, Illumina NovaSeq, Oxford Nanopore, sugarcane, Brazil, soil microbiome

## Abstract

We report the complete genome sequence of *Micrococcus luteus* TG3.30, isolated from sugarcane soil in Brazil. The 2.58 Mb genome (GC 72.92%) was assembled from hybrid Illumina–Nanopore sequencing with 99.96% completeness, providing a high-quality resource for taxonomic and comparative genomic studies of this versatile actinobacterium.

## ANNOUNCEMENT

*Micrococcus luteus* is a gram positive, non-motile, strictly aerobic coccus (family Micrococcaceae, order Actinomycetales) found in soil, water, air, and skin microbiota. It possesses a high GC-content genome and notable metabolic versatility (1–8). Traditionally seen as a harmless saprophyte, *M. luteus* is now recognized for diverse roles in environmental, industrial, medical, and agricultural contexts.

Here, we report the complete genome sequence of *M. luteus* strain TG3.30, isolated in 2017 from sugarcane plantation soil (Usina Açucareira Virgolino de Oliveira S/A, José Bonifácio, SP, Brazil; 21°5′12.149″ S, 49°55′13.714″ W). Isolation and purification by serial streaking followed Egea et al. (2014, 2017) (9, 10). For sequencing, the strain was grown in LB medium (12 h, 30°C, 150 rpm). Identification was confirmed by 16S rRNA Sanger sequencing (GenBank PV989629; primers 27F/1492R) via NCBI BLASTn (E-value < 1e–20, identity > 97%).

Strain TG3.30 was cultured in Lysogenic Broth (tryptone 1%, NaCl 0.05%, yeast extract 0.5%) for 12 h, harvested by centrifugation (15,000×g, 15 min, 4°C), and DNA was extracted using the Zymo Quick-DNA Fungal/Bacterial Miniprep Kit. The same DNA sample was used for both sequencing techniques by Eurofins Genomics. Illumina libraries were prepared using a fragmentation–ligation (NEBNext Ultra II FS DNA Library Prep Kit) with TruSeq adapter sequences (Eurofins Genomics proprietary protocol) and sequenced on a NovaSeq X platform (2×150 mode). The Oxford Nanopore (ONT) library used the Rapid Barcoding Kit 24 V14 (SQK-RBK114.24) without size selection and was sequenced on an R10.4.1 (FLO-MIN114) flow cell. ONT base calling used Dorado (high-accuracy model dna_r10.4.1_e8.2_400bps_sup@v4.3.0).

Nanopore reads were quality-filtered with Filtlong v0.2.1 (11) and assembled *de novo* with Flye v2.9.3 (12). A hybrid assembly was generated using Unicycler v0.5.0 (13) and polished with Medaka v1.8 (14) and Pilon v1.24 (15). Reads were mapped using Minimap2 v2.24 (16). Assembly quality was assessed via QUAST v5.2 (17) for structural metrics, CheckM2 v1.0.1 (18) for completeness/contamination, and Mash v2.3 (19) for similarity-based QC. Taxonomic assignment used CheckM2 v1.0.1 (18).

**Fig 1:**
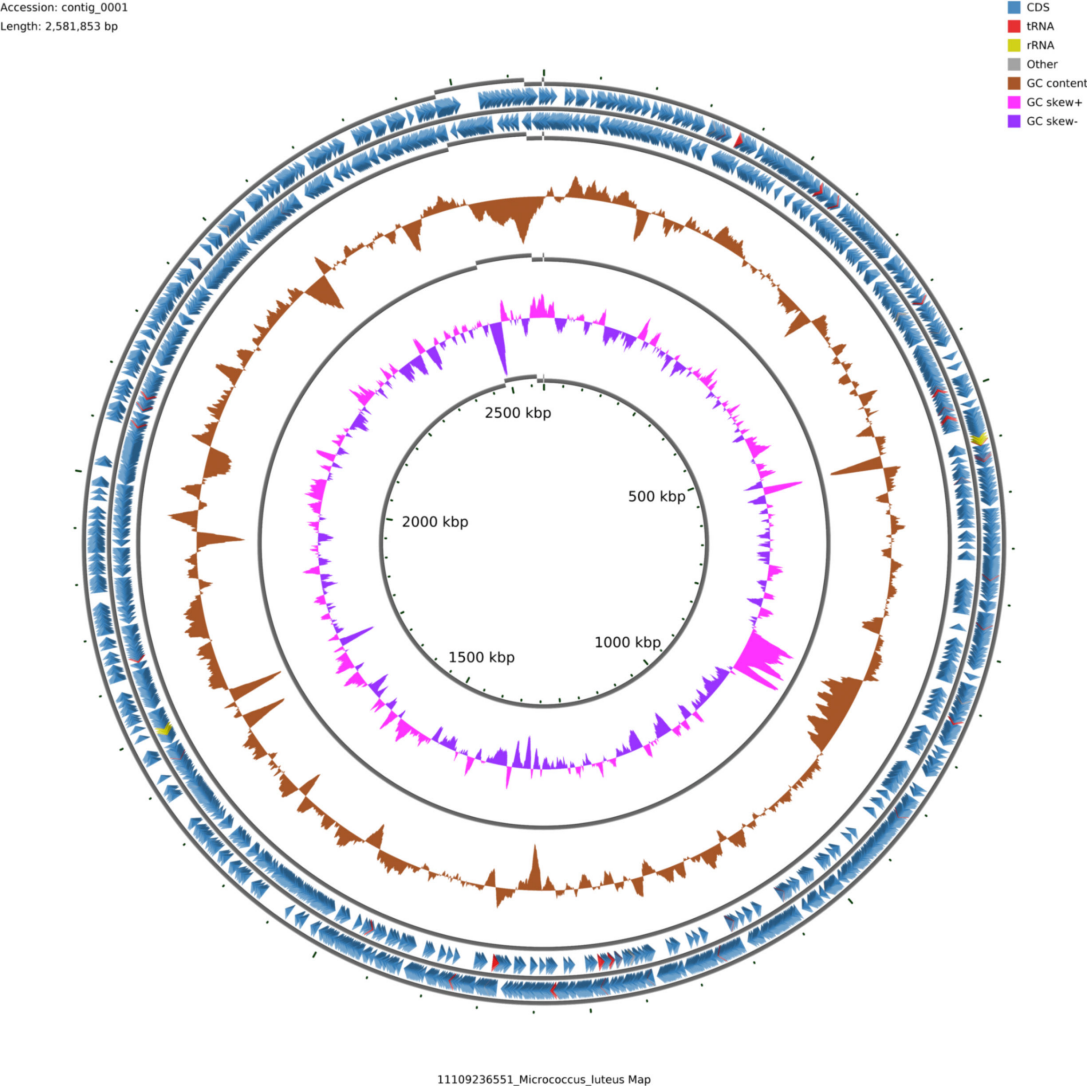
Circular genome map of M. luteus TG3.30. The two outer rings show coding regions (forward/reverse strands), including tRNA, rRNA, and other annotated features. The GC content track illustrates deviations from the genome average, while GC skew identifies replication origin and terminus regions. The circular genome representation was generated using CGView v3.0 (20) with default parameters*.*

Illumina sequencing produced 4,954,823 paired-end reads (1.49 Gb). ONT sequencing generated 5,019 filtered reads (34.21 Mb; ~13× coverage), with mean/median quality scores of 19.6/21.9. ONT metrics: mean 6,817 bp, median 4,938 bp, N50 10,377 bp, and max 44,404 bp. The hybrid assembly yielded a 2,581,853 bp genome (72.92% GC) in four contigs (2,483,961 bp, 47 kb, 32 kb, 18 kb) with an N50 of 2.48 Mb (Fig. 1). Completeness was 99.96% with 0.08% contamination. Taxonomy confirmed 97.05% identity to *Micrococcus aloeverae*, *M. luteus*, and *Micrococcus* sp. Annotation identified 2,354 CDSs, 48 tRNAs, 6 rRNAs, 2 ncRNAs, and 11 pseudogenes.

All software tools were executed with default parameters unless stated otherwise.

## Data Availability

This Whole Genome Shotgun project has been deposited in GenBank under the accession no. JBPGVW010000000 (21). The version described in this paper is the first version. Biosample: SAMN49445049, Bioproject: PRJNA1279181, Illumina Sequence Read Archive (SRA): SRR36594263, Nanopore SRA: SRR36252807.
